# Supplementary material for: In Silico Modeling and Characterization of Epstein–Barr Virus Latent Membrane Protein 1 Protein
Source: ACS Omega. 2024 Dec 2;9(50):49422–31. doi: 10.1021/acsomega.4c06868 (PMC11656244; doi:10.1021/acsomega.4c06868)
Supplement: Supplementary file 1 — ao4c06868_si_001.pdf [file ao4c06868_si_001.pdf]

In silico modelling and characterization of Epstein–Barr virus LMP1 protein

Dayang-Sharyati D.A. Salam[1], Kavinda Kashi Juliyan Gunasinghe[1], Siaw San Hwang[1], Irine Runnie Henry Ginjom[1], Xavier Chee Wezen[1,3]*, Taufiq Rahman[2]*

[1]Faculty of Engineering, Computing and Science, Swinburne University of Technology Sarawak, Kuching 93350, Malaysia.

[2]Department of Pharmacology, University of Cambridge, Cambridge CB2 1PD, United Kingdom.

[3]Department of Biochemistry, Yong Loo Lin School of Medicine, National University of Singapore, Singapore 117596

Corresponding authors:

* Email: mtur2@cam.ac.uk ;

* Email: xchee@swinburne.edu.my

Supporting Information

Table S1 Ab initio docking results for LMP1 predicted structure.

Model No.	No. of Subunits	Interface Area (in Angstrom ^{2})	Docking Score
1	2-mer	2279.7	2350.543
2	2-mer	2173.8	2206.530
3	2-mer	1804.2	2103.901
4	5-mer	7891.9	1012.986
5	3-mer	3869.4	914.557

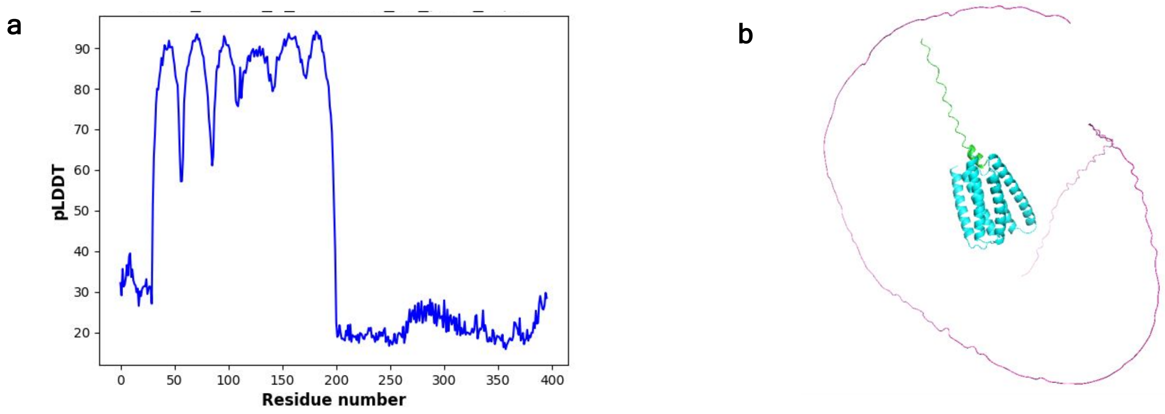

Figure S1 (a) Per-residue confidence score for LMP1 model 3 **(b)** AlphaFold2 LMP1 model 3 image

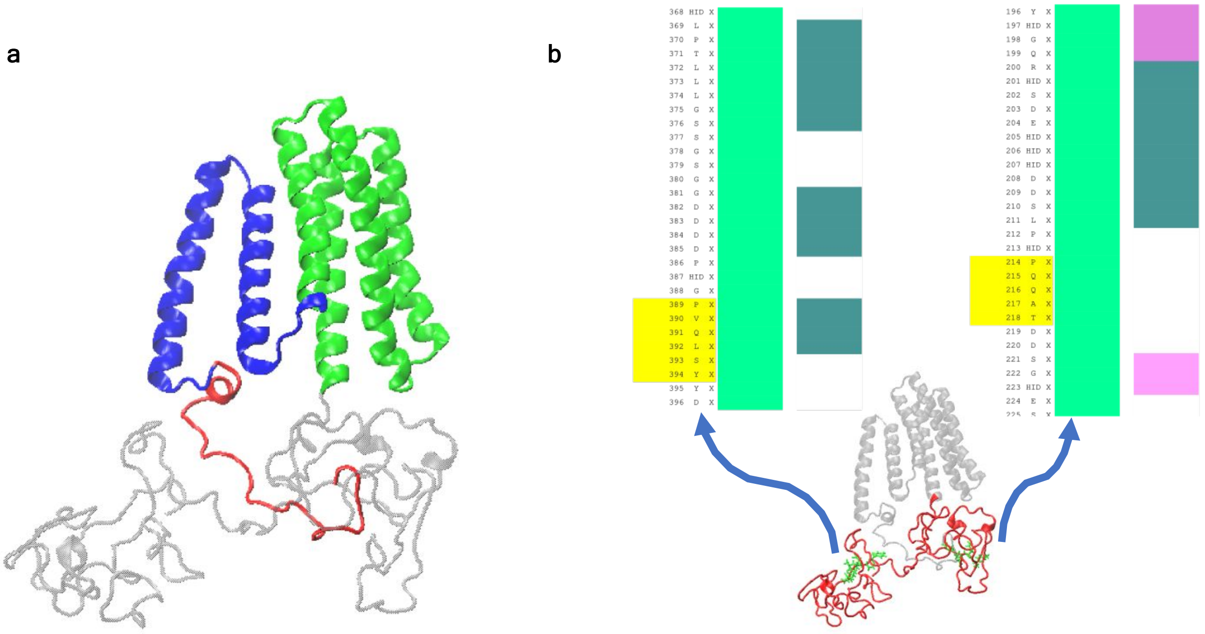

Figure S2 (a) The molecular image of TMD3-6 (green colour) and TMD1-2 (blue colour) responsible for the oligomerisation of the LMP1 protein. The red-colour chain represents the N-terminal domain of the LMP1 protein. **(b)** The CTAR1 and CTAR2 domains activate the cells.

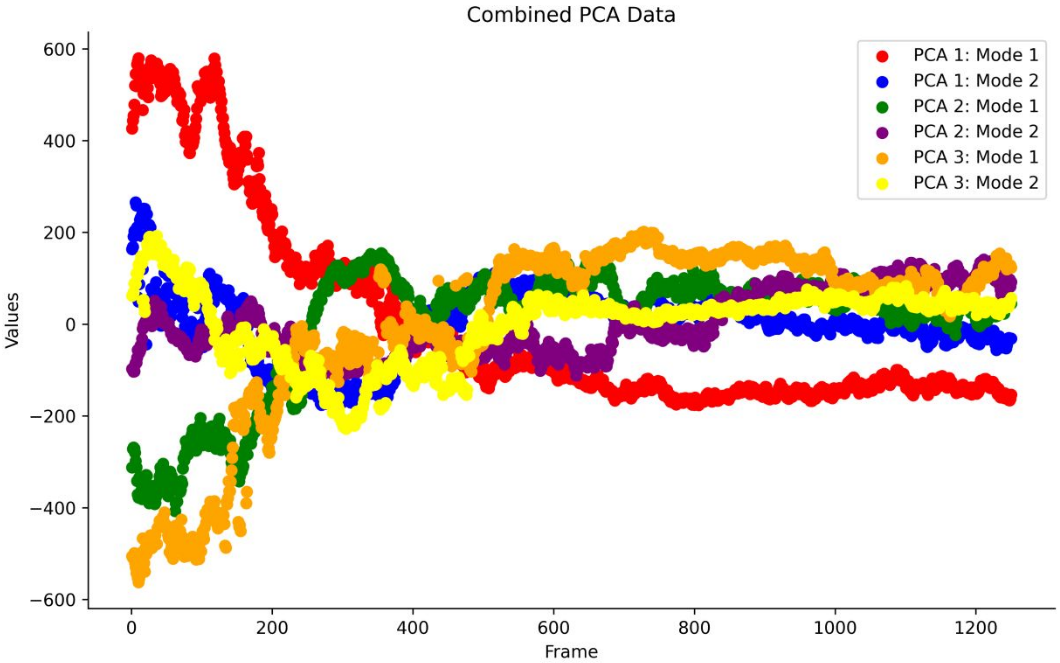

Figure S3 Graph shows combined PCA data for all the triplicate runs with the values on the y-axis and frame on the x-axis.
